# 

**Published:** 1882-06

**Authors:** 


### CONTENTS:

ART. I.—Treatment of the Teeth with Dead or Dying Pulps, and Alveolar Abscess. By Dr. H. H. Townsend.........49
ART. II.—Origin, Definition and Division of Tissues. By Dr. C. Heitzman.........54
ART. III.—Operative Dentistry. By Geo. S. Mills, D. D. S.........70
ART. IV.—What is Facial Neuralgia? By Parsons Shaw, D. D. S.........78
ART. V.—Removal of Naso-Pharyngeal Polypus. By Dr R. F. Weir.........83
Pennsylvania State Dental Society.........86
Pittsburg Dental Association.........87
American Dental Association.........87
National Dental Association.........88
Southern Dental Association.........88

### EDITORIAL, ETC.

The New Building of the Dental Department of the University of Maryland.........88
The Baltimore College of Dental Surgery.........89
Southern Dental Association Meeting.........91

### BIBLIOGRAPHICAL.

Manual of Dental Surgery and Pathology. By Alfred Coleman, L. R C. P Revised by Dr. Stellwagen.........92
A Manual of Dental Anatomy. By Charles Tomes, M. A., F. R. S. Sec'd Edition...93

### MONTHLY SUMMARY.

Dental Surgeons in the Army and Navy.........93

Therapeutic uses of Nitrite of Amyl.........94

A New Fungus.........95

Therapeutic Effects of Oxygen.........96

A Triumph of Dentistry.........96

College Examination for the Degree of Doctorate.........96

